# Quasi-Classical Trajectory Study of the CN + NH_3_ Reaction Based on a Global Potential Energy Surface

**DOI:** 10.3390/molecules26040994

**Published:** 2021-02-13

**Authors:** Joaquin Espinosa-Garcia, Cipriano Rangel, Moises Garcia-Chamorro, Jose C. Corchado

**Affiliations:** Departamento de Química Física and Instituto de Computación Científica Avanzada, Universidad de Extremadura, 06071 Badajoz, Spain; ciprira@unex.es (C.R.); moises@unex.es (M.G.-C.); corchado@unex.es (J.C.C.)

**Keywords:** potential energy surface, deep wells, kinetics study, dynamics study, QCT calculations

## Abstract

Based on a combination of valence-bond and molecular mechanics functions which were fitted to high-level ab initio calculations, we constructed an analytical full-dimensional potential energy surface, named PES-2020, for the hydrogen abstraction title reaction for the first time. This surface is symmetrical with respect to the permutation of the three hydrogens in ammonia, it presents numerical gradients and it improves the description presented by previous theoretical studies. In order to analyze its quality and accuracy, stringent tests were performed, exhaustive kinetics and dynamics studies were carried out using quasi-classical trajectory calculations, and the results were compared with the available experimental evidence. Firstly, the properties (geometry, vibrational frequency and energy) of all stationary points were found to reasonably reproduce the ab initio information used as input; due to the complicated topology with deep wells in the entrance and exit channels and a “submerged” transition state, the description of the intermediate complexes was poorer, although it was adequate to reasonably simulate the kinetics and dynamics of the title reaction. Secondly, in the kinetics study, the rate constants simulated the experimental data in the wide temperature range of 25–700 K, improving the description presented by previous theoretical studies. In addition, while previous studies failed in the description of the kinetic isotope effects, our results reproduced the experimental information. Finally, in the dynamics study, we analyzed the role of the vibrational and rotational excitation of the CN(v,j) reactant and product angular scattering distribution. We found that vibrational excitation by one quantum slightly increased reactivity, thus reproducing the only experimental measurement, while rotational excitation strongly decreased reactivity. The scattering distribution presented a forward-backward shape, associated with the presence of deep wells along the reaction path. These last two findings await experimental confirmation.

## 1. Introduction

Very exothermic reactions with stable intermediate complexes in the entrance and exit channels represent a true theoretical challenge, both in terms of the electronic structure calculations of the species involved and regarding the kinetics and dynamics tools used in their study. This problem obviously increases with molecular size. The gas-phase reaction of the cyanogen radical with ammonia is an interesting example of this kind of neutral–neutral reaction, which presents large rate constants and shows an inverse temperature dependence at T < 300 K, where the rate constant increases by a factor of 17 when the temperature decreases from 198 to 25 K [1]. This behavior is associated with the presence of a stable intermediate complex in the entrance channel. Apart from its fundamental interest, knowledge of this reaction is important to understand the chemistry of dense interstellar clouds, and it plays a noticeable role in the chemistry of the Titan atmosphere [1].

While the title reaction has been experimentally widely studied [2], theoretical studies, in which mainly low computational calculations are used in the description of the reactive system, are scarce [3,4,5,6,7]. The first experimental measurements of the rate constants were performed by Boden and Thrush in 1968 at 687 K [8], and an extensive literature review can be found in the National Institute of Standards and Technology (NIST) kinetics database [2]. While the first review performed by Baulch et al. [9] in the temperature range of 300–700 K reported a rate constant k(T) of 1.66.10^−11^ cm^3^ molecule^−1^s^−1^, which was invariant with temperature, Sims and Smith [10] reported a rate constant k(T) of 1.52.10^−11^ exp(1.50[kJ·mol^−1^]/RT) cm^3^ molecule^−1^s^−1^, in the temperature range of 294–761 K, with a slight variation with T. A similar tendency was reported by Yang et al. [1] in the temperature range of 221–740 K, with a k(T) of 10^−11.02^ exp(254/T) cm^3^ molecule^−1^s^−1^. However, when the temperature range was extended to lower values of 25–295 K and a k(T) of 2.77.10^−11^ (T/298 K)^−1.14^ cm^3^ molecule^−1^s^−1^, a strong increase in the rate constant by a factor of 17 was found when the temperature decreased [11]. Besides the rate constants at different temperatures, the effects of the deuteration of ammonia, ND_3_ [6] and of the CN(v) vibrational excitation [10] were experimentally reported. Theoretically, this reaction has been less studied, in general using low-level computational methods. To explain the kinetics of the title reaction, the first theoretical calculations [3,6] were based on capture methods [12,13], where the rate constants were related with long-range potentials between reactants. However, it is known that capture theories tend to overestimate rate constants [14]. Meads et al. [6] performed a combined experimental (296 K) and theoretical study on the title reaction and its deuterated isotope, CN + NH_3_/ND_3_. The theoretical rate constants were obtained using a capture method based on ab initio calculations with the fourth-order Møller–Plesset perturbation method (MP4) on optimized geometries at the MP2 level, in the temperature range of 200–500 K. They concluded that the agreement with experiment was tolerable, but the kinetic isotope effect (KIE) was not reproduced. Thus, these authors reported an experimental KIE (296K) NH_3_/ND_3_ of 2.00, in contrast with the theoretical value (300 K) of 0.95. Later, Faure et al. [4,5] showed that long-range potentials alone are not adequate to describe this system and performed quasi-classical trajectory (QCT) calculations on modified potentials using ab initio calculations at the coupled cluster single-double and perturbative triple level CCSD(T) on optimized geometries at the CCSD level. In the temperature range of 25–300 K, they found a flat temperature dependence of the rate constants, in strong disagreement with the inverse temperature dependence experimentally observed. In 2009, in—to the best of our knowledge—the last published paper on the title reaction, Talbi and Smith [7] calculated the minimum energy path at the CCSD(T) theoretical level and obtained the rate constants (25–716 K) using the two transition-state model of Klippenstein et al. [15,16,17,18] They found that the theoretical rate constants reproduced the experimental evidence in this wide temperature range, although they concluded that, due to the approximations used in the theoretical tools, agreement was perhaps slightly fortuitous. Finally, from the extensive experimental and theoretical evidence, the title reaction presents a complex reaction mechanism, which proceeds via intermediate species in the entrance and exit channels,

CN(X^2^Σ^+^) + NH_3_ ↔ RC → SP → PC → HCN + NH_2_ (1)

where RC, SP and PC mean, respectively, the reactant complex, saddle point and product complex. This reaction presents a large exothermicity of ΔH_r_ (298 K) = −18.47 [19], −18.93 [20] kcal mol^−1^, with a “submerged” barrier (i.e., the energy below the reactants) [7] of −2.60 kcal·mol^−1^. In addition, Talbi and Smith [7], based on theoretical calculations, concluded that the alternative path leading to the NCNH_2_ + H products is unlikely and should be discarded.

Despite the theoretical effort undertaken to analyze the title reaction, some issues remain to be dealt with. For instance, (i) the development of a global potential energy surface describing all stationary points, (ii) the use of higher level ab initio calculations to describe the reactive system, (iii) the use of adequate theoretical tools to describe this complex mechanism with deep wells in the entrance and exit channels and the existence of a “submerged” transition state, and finally, (iv) the analysis of some dynamics properties which could to help to understand this reactive system.

The paper is organized as follows. In Section 2, we detail the development, for the first time, of an analytical full-dimensional potential energy surface (PES), named PES-2020, which is a valence bond/molecular mechanics (VB/MM) functional model based exclusively on high-level ab initio calculations. On this surface, a kinetics and dynamics analysis was performed; the computational details of the tools used and quasi-classical trajectory (QCT) calculations are shown in Section 3. In Section 4, the kinetics results are analyzed, discussed and compared with the available experimental information. Section 4 also includes an analysis and discussion of the dynamics results. Some of these results cannot be compared with other theoretical or experimental results because they are not available. Consequently, some dynamics results presented in the present paper represent the first studies on this issue, and so they are predictive in nature and must be confirmed by future experiments. Finally, in Section 5, we summarize the main conclusions.

## 2. Global Potential Energy Surface

The full-dimensional PES-2020 is a fit for high-level ab initio calculations. All stationary points, reactants, products, intermediate species and saddle points were optimized and characterized (by vibrational frequency calculations) with the explicitly correlated coupled cluster method with single, double and triple excitations, CCSD(T)-F12, using the correlation-consistent polarized double-zeta basis set, aug-cc-pVDZ. In brief, the CCSD(T)-F12a/aug-cc-pVDZ level was named Level 1. These calculations were performed with the ORCA code [21]. The minimum energy path, MEP, which connects reactants and products through the saddle point, was obtained with a lower level due to the computational cost, CCSD(T)/6-311++G(d,p), which was named Level 2. In addition, Hessians were calculated at 50 points along the MEP to describe the reaction valley. The computational effort was therefore equivalent to about 16,000 energy calculations (18 coordinates corresponding to six atoms, and 18 squared results in 324 energy calculations per point times 50 points along the MEP). Note that, in this barrierless reaction, the tunneling effect is negligible, so the description of zones far from the reaction valley by using electronic structure calculations is of little importance. These calculations were performed with the GAUSSIAN code [22].

Following the experience of our group using the VB/MM method to develop potential energy surfaces in polyatomic systems, the present surface is based on our knowledge of surfaces [23,24,25,26,27] describing several hydrogen abstraction reactions with ammonia, F(^2^P) + NH_3_, H + NH_3_, O(^3^P) + NH_3,_ Cl(^2^P) + NH_3_ and OH + NH_3_, because similar stretching and bending motions are involved; obviously, the necessary modifications were implemented to adapt them to the present study. Given that a term-by-term description of all functional forms of the present PES-2020 surface has been given in previous studies about reactions with ammonia, we provide here only a brief summary to avoid repetition. The global surface is developed as the sum of two terms,

(2) $$V=V_{stretch}+V_{bending}$$

where the first term is a valence bond stretching term, which describes the three equivalent N–H bonds in ammonia and is developed as the sum of three London-Eyring-Polanyi terms,

(3) $$V_{stretch}=\overset{3}{\underset{i=1}{\sum }}V_{3}\left(R_{NH_{i}},R_{NC},R_{H_{i}C}\right)$$

where $H_{i}$ represents one of the three ammonia hydrogens and R is the distance between the two subscript atoms. The $V_{3}$ potentials are developed as a function of singlet and triplet dissociation energies, $D_{XY}^{1}$ and $D_{XY}^{3}$, equilibrium bond distances, $R_{XY}^{eq}$, and Morse parameters, $a_{XY}$. In addition, switching functions are used to relax from ammonia to the amidogen radical when the reaction progresses from reactants to the product. Therefore, *V_stretch_* depends on 14 adjustable parameters.

*V_bending_* is a molecular mechanics potential term describing bending motions, defined as the sum of three harmonic terms (one for each equivalent bond angle in ammonia),

(4) $$V_{bending}=\frac{1}{2}\;\overset{3}{\underset{i=1}{\sum }}\overset{3}{\underset{j=i+1}{\sum }}k_{ij}^{0}k_{i}k_{j}{\left(\theta_{ij}-\theta_{ij}^{0}\right)}^{2}$$

where $\theta_{ij}^{0}$ is the reference angle in ammonia and $k_{ij}^{0}$ and $k_{i}$ are force constants. As with the $V_{stretch}$ term, switching functions are used to allow this angle to relax from its value in ammonia to its value in products. So, the $V_{bending}$ terms depend on 16 parameters. Finally, two new terms are included to take into account the N–H_i_ bond in reactants which evolve to H-CN in products: a Morse function to describe the N–H*_i_* bond, and a harmonic bending term describing the collinear H_i_-C-N bending mode in products. Thus, three new parameters are used. The switching functions in all terms are devised to allow smooth changes from reactants to products while the reaction evolves. Hyperbolic tangent functions are used for this as they show the desired mathematical properties of being smooth and differentiable in the region of interest. In total, the new PES-2020 surface depends on 33 adjustable parameters and is symmetrical with respect to the permutation of the three equivalent hydrogens in ammonia. These features give great flexibility to the functional form and keep the potential physically intuitive; i.e., based on stretching and bending terms. This is an advantage of the VB/MM method for developing surfaces in polyatomic surfaces as compared with more computationally demanding methods, which use thousands of high-level ab initio calculations. Note that, in the present version, only numerical gradients are available for PES-2020.

Once the functional form is developed, the 33 adjustable parameters are fitted to the previously calculated ab initio information, which is used as an input. In a first approximation, by using the least-square method (where our own code [28] is used, which includes energies, gradients and Hessians at all stationary points and along the reaction path, which are conveniently weighted) with all 33 adjustable parameters simultaneously, the process was unsuccessful. Thus, by taking advantage of the fact that most parameters are related to chemical properties, such as bond lengths, dissociation energies, barrier height, etc., the fitting problem was divided into four iterative steps: (i) first, we focused on the properties of the reactants and products: geometry, energy and harmonic vibrational frequencies; (ii) next, we analyzed the saddle point properties, geometry, barrier height and vibrational frequencies, with special attention paid to the imaginary vibrational frequency, which was related to the width of the barrier; (iii) in this reaction, the location and characterization of the wells in the entrance and exit channels was very important for the posterior kinetics and dynamics analysis. This fitting step was especially difficult because, given the high exothermicity of the reaction, the reactant complex was very close to the reactants in the reaction path, and all changes in the parameters related to reactants affected the description of this complex. To this end, we found a compromise between time, efficacy and accuracy; iv) finally, we aimed to reproduce the minimum energy path and the reaction valley, which were related with a greater or lesser fall from the saddle point to reactants and products, and this behavior had dynamic consequences, for instance, on product energy redistribution. As previously noted, this was an iterative process which was repeated until a reasonable convergence was achieved with the ab initio input information. Obviously, given that 33 adjustable parameters were involved, the fitting process was very time-consuming. The full-dimensional PES-2020 can be obtained upon request from the authors, and will be published soon in the POTLIB library [29].

## 3. Kinetics and Dynamic Tools

Based on the global PES-2020, in the kinetics and dynamics studies, QCT calculations were performed with the VENUS code [30,31], using standard expressions [32,33]. The Monte Carlo approach implemented in VENUS was used in the selection of the initial scattering parameters (spatial orientation, impact parameters and vibrational phases). The integration step was 0.01 fs to ensure the stability of the trajectories, while trajectories were initiated and stopped with reactants and products, respectively, separated by 15 Å, to ensure negligible molecular interactions. Several temperatures in the range of 25–700 K were selected for the kinetics analysis, where the maximum impact parameter, $b_{max}$, was obtained at each temperature, ranging from 16.0 Å at 25 K to 12.0 Å at 700 K. The reactant vibrational and rotational energies and the relative translational energy were chosen by thermal sampling at each temperature, and 10^5^ trajectories were run at each temperature. In these conditions, the reaction cross section, $\sigma_{r}\left(T\right)$, is obtained by the expression

(5) $$\sigma_{r}\left(T\right)\;=\;\pi b_{max}2\;N_{r}/N_{T}$$

where $N_{r}$ and $N_{T}$ correspond to the number of reactive trajectories and the total number of trajectories run at each temperature, with the estimated error given by

(6) $$\Delta \sigma_{r}\left(T\right)=\sigma_{r}\sqrt{\frac{N_{T}-N_{r}}{N_{T}N_{r}}}$$

and the rate constant given by

(7) k(T)=(8kBTπμ)12σr(T)

where *µ* and *k_B_* are the reduced mass and the Boltzmann constant, respectively. At all temperatures, given the high reactivity and the great number of trajectories run, the errors are always ≤5%.

The QCT method, given its classical nature, suffers from a serious limitation: the non-consideration of quantum effects, such as tunneling and zero-point energy (ZPE). In this case, with a “submerged” barrier, the title reaction can be considered as a barrierless reaction, and so, a priori, the tunneling contribution is negligible. However, the ZPE violation problem, corresponding to reactive trajectories finishing with products’ vibrational energy below their respective ZPE, is a serious drawback. Different alternatives have been proposed to solve this problem, though none are definitive [34,35,36,37,38]. In order to minimize this defect, we used two passive approaches. In the first approach, we only considered reactive trajectories in which each product, HCN and NH_2_, finished with a vibrational energy above its ZPE. This is named the double ZPE approach (DZPE). However, this is a very drastic approach, and Schatz et al. [39,40] suggested that the ZPE correction should be applied only to the HCN product; i.e., the newly formed bond. This constraint is named ZPE-HCN. In a previous study combining QCT and QM (quantum mechanics) [41] on the H + NH_3_ reaction, we found that this last constraint method presented the best agreement with the QM results. Thus, based on this experience, we chose the ZPE–HCN approach in the present paper.

## 4. Results and Discussion

### 4.1. Accuracy of the Fitting Process; Self-Consistency Test

**Figure 1:**
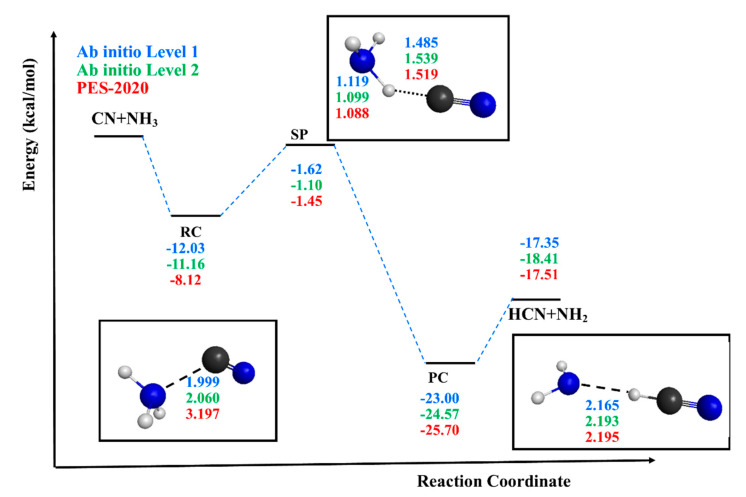
Classical energy diagram of the stationary points for the CN + NH_3_ reaction. First entry, Level 1, CCSD(T)-F12a/aug-cc-pVDZ; second entry, Level 2, CCSD(T)/6-311++G(d,p); and third entry, potential energy surface (PES)-2020. As insets, we include the optimized geometries of the reactant complex, RC2, saddle point and product complex. Energy in kcal mol^−1^ and distances in Å. Blue ball: N atom; gray ball: H atom; black ball: C atom.

The geometric, energetic and vibrational information shown in Figure 1 and Table 1; Table 2, describing all stationary points, represents a first test of the accuracy of the new PES-2020, as compared with the ab initio information used as input. We begin by analyzing the properties of the reactant and products. In general, PES-2020 reproduces the ab initio information used as input. So, the ZPE differences are lower than 0.5 kcal mol^−1^, and PES-2020 reproduces the enthalpy of reaction at 298 K obtained at Levels 1 and 2, and they all reproduce the experimental evidence, ΔH_r_ (298 K) = −18.47 [19], −18.93 kcal mol^−1^ [20], taking into account the experimental error of ±2 and thus showing a very exothermic reaction. As noted, the saddle point has a “submerged” barrier, and therefore its location presents difficulties. Levels 1 and 2 have barriers of −1.62 and −1.10 kcal mol^−1^, with imaginary vibrational frequencies of 878 i and 697 i cm^−1^; i.e., different curvatures of the reaction path. In general, these results agree with previous theoretical results [7] obtained at a similar ab initio level. In fact, Talbi and Smith [7] also obtained a submerged transition state with an imaginary frequency of 846 i cm^−1^. PES-2020 reproduced these results, presenting an intermediate situation, with a barrier of −1.45 kcal mol^−1^ and 810 i cm^−1^ of imaginary frequency. In this reaction, the broken bond, N–H’, was smaller than the new formed bond, H´–C, and this behavior was reproduced with PES-2020.

Undoubtedly, the largest differences were found in the location and characterization of the intermediate complexes. As previously noted in the fitting process (Section 2), this search represented a true challenge, and we finished the process with a compromise between time, efficacy and accuracy. So, PES-2020 gave a first approximation to the description of these deep wells in the entrance and exit channels, but the differences with respect to the ab initio information were more important than in the other stationary points. However, taking into account the fact that the kinetics and dynamics description of the title reaction simulated the experimental evidence reasonably (see Results section), we considered that the description of these very stabilized complexes was sufficiently good to study this reaction. In our previous studies on the reactions of free radicals (X = Cl, OH) with ammonia [26,42], Cl(^2^P) + NH_3_ and OH(X^2^Π) + NH_3_, we found several complexes in the entrance channel. A priori, given the symmetry of ammonia, with three equivalent hydrogens, three complexes could be formed: (i) the free radical approaches one H atom of ammonia, with an orientation of X···H–NH_2_ and denoted RC1; (ii) the free radical approaches the nitrogen atom of NH_3_ with the three hydrogen atoms on the opposite side, with an orientation of X···NH_3_ and denoted RC2; and finally (iii) the free radical approaches the N atom of ammonia, but now the three hydrogens are on the same side, with an orientation of X···H_3_N and denoted RC3. In the case of the reaction with chlorine [42], Cl(^2^P) + NH_3_, all three complexes were located and characterized both by ab initio calculations and by the analytical PES developed for this system, PES-2010 [27]. RC1 and RC3 were characterized as hydrogen-bonded complexes. With respect to the RC2 complex, the analysis of the corresponding molecular orbitals at the CCSD(T)/-cc-pVTZ ab initio level showed that the large stability of this complex was related with the existence of a two-center three-electrons (2c-3e) hemibond complex. In the case of the reaction with the hydroxyl radical, OH(X^2^Π) + NH_3_, only the RC1 and RC3 orientations were located with ab initio calculations and with the analytical global PES developed for this system, PES-2012 [26]. In the reaction studied here, CN(X^2^Σ^+^) + NH_3_, we performed a similar analysis of the possible complexes in the entrance channel. At the ab initio Levels 1 and 2, only the RC2 complex was located and characterized, stabilized by 12.03 and 11.16 kcal mol^−1^, respectively, with respect to the reactants. From the bibliographic search, only one theoretical study [7] located the RC2 complex, while the RC1 and RC3 complexes were not reported. In the comparison with the results obtained with PES-2020, we found that all three complexes were located and characterized as true minima, stabilized with respect to the reactants by 7.24 (RC1), 8.12 (RC2) and 7.00 (RC3) kcal mol^−1^, all of them presenting Cs symmetry. In the RC2 reactant complex, the CN radical approached the N atom in ammonia with a C–N distance of about 2.0 Å at Levels 1 and 2, while PES-2020 presented a larger distance of 3.197 Å. As a consequence, this RC2 was more stabilized using Levels 1 and 2 (differences of 3–4 kcal mol^−1^). However, when the ZPE was considered—i.e., enthalpies at 0 K were compared—these differences decreased to 1.0–1.5 kcal mol^−1^. Note that only this common RC2 complex appears in Table 2 and Figure 1. These electronic structure calculations show that these complexes in the entrance channel proved to be very elusive on the potential surface at each level, emphasizing the theoretical difficulty in the description of this reactive system. In order to analyze this behavior, several factors were analyzed: (i) electronic structure calculations. In addition to Levels 1 and 2, Hartree–Fock/6-31G(d,p), MP2/6-31G(d,p) and two DFT methods, Minnesota functional M062X/6-311++G(d,p) and modified Perdew–Wang functional MPW1K/aug-cc-pVTZ were used to locate these reactant complexes. In all cases, starting from different orientations, only the RC2 complex was found. (ii) The multi-reference character of the wave function for the CN free radical. The T1 diagnostic [43] is a measure of this multi-reference character, and a large value of T1 (T1 > 0.02) is an indication that this effect could be important. For CN, RC2 and SP, this value was, respectively, 0.0925, 0.0436 and 0.0462, all of which were larger than 0.02. This behavior shows that single-reference electron correlation methods could be unsuitable and multi-reference methods could be necessary. A multi-reference study is beyond the scope of the present work, but we have performed a simple approximation to understand the importance of this problem for the stability of this complex by performing a CASSCF study. The complete active space (CAS) was chosen including the π and π* molecular orbitals of CN and the molecular orbitals involving contributions from the CNN fragment. Thus, eight OM and 11 electrons were used, CASSCF(8,11), with the 6-31G(d,p) basis set. Again, starting from different orientations in the reactant complex, only the RC2 was found. So, it seems that this was not the decisive factor. (iii) Finally, the electronegativity of the attacking free radical was analyzed. Using ab initio calculations, in the reactions with Cl(^2^P), all three reactant complexes were located, while in the reaction with OH(^2^Π), only the RC1 and RC3 complexes were located, and in the studied reaction, only the RC2 was obtained. RC1 and RC3 are hydrogen-bonded complexes which are related to the large electronegativity of the Cl(^2^P) and oxygen in the OH(^2^Π) radical. The CN radical, however, presents the lowest electronegativity (C or N), and therefore the hydrogen bond complexes are weaker or even unstable. In the case of the RC2 complex, we suppose that a 2c-3e hemibond complex, H_3_N···CN, similar to the complex with the chlorine atom is formed.

In the exit channel, only one product complex (PC) was located. The PES-2020 geometric description improved with respect to the RC complex, the N···H distance obtained at the ab initio levels, at 2.165–2.193 versus 2.195 Å, but the energetic description was still not as good as in other stationary points. We suppose that this difference in the PC had little influence on the dynamics of the reaction, because the high exothermicity and the kinetic energy of the products were so large that they quickly separated. In summary, in spite of this quantitative disagreement, PES-2020 simulated the difficult topography of the complete system reasonably well, with deep wells in the entrance and exit channels. Finally, note that this good agreement in reactants, products and saddle point and reasonable agreement in the intermediate complexes is merely a test of self-consistency, because the ab initio information was used in the fitting process.

**Figure 2:**
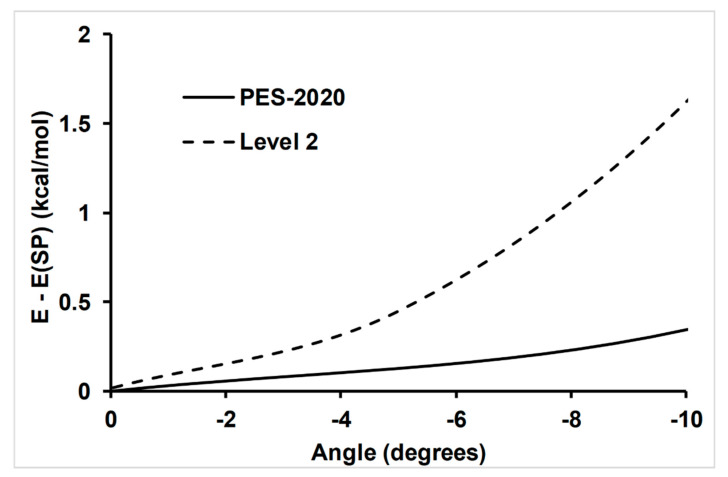
Dependence of energy on the bonding angle in the saddle point, N–H´–C. The equilibrium geometry at each calculation is taken as a reference (zero level). The other internal coordinates were fixed at the values in the respective saddle point. PES-2020, solid line; and Level 2, CCSD(T)/6-311++G(d,p), dashed line.

The second test in this comparison was related to the dependence of the N–H´–C bonding angle in the saddle point (Figure 2), which was related with the rotational distribution of the HCN product. So, tighter saddle points were related to colder rotational distributions. PES-2020 presented a more attractive potential than the ab initio calculations at Level 2, and so one should expect hotter rotational distributions.

**Figure 3:**
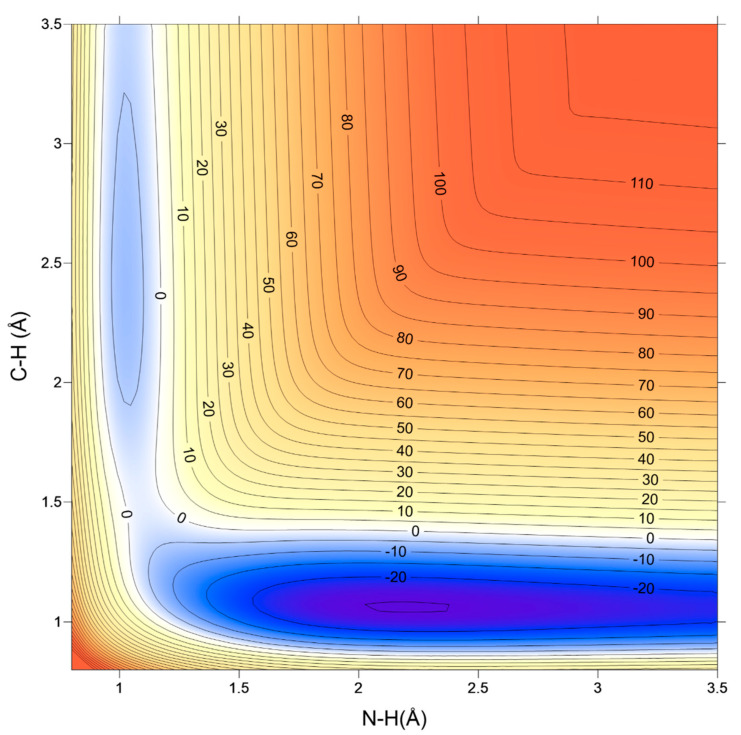
Schematic 2D representation of the PES-2020 surface describing the reactive system of CN + NH3 as a function of the broken and formed bonds, N–H and H–C, respectively, in Å. The other internal coordinates were fixed at the values in the saddle point. Energies are shown in kcal·mol^−1^. The reactant and product complexes appear clearly in the entrance (upper-left zone) and exit (lower-right zone) channels in the plot.

Finally, a complete representation of the topography of the new PES-2020 surface is shown in Figure 3, where the N–H´ broken and H´–C formed bonds (in Å) are represented. This barrierless and very exothermic reaction shows a smooth and continuous behavior without spurious minima, even when describing zones for which no ab initio information is available. In addition, it is free of unphysical features and differentiable. The deep wells in the entrance and exit channels are also clearly visible. Note that, due to the fact that the distances plotted are N–H´ and H´–C, the complex on the entrance channel corresponds to the RC1 orientation. The PES-2020/ab initio differences are measured using the root-mean-square error (RMSE), which is given by the expression

(8) $$RMSE=\sqrt{\overset{N_{data}}{\underset{i=1}{\sum }}\frac{{\left(E_{PES}-E_{ab\;initio}\right)}^{2}}{N_{data}}}$$

where $E_{PES}$ and $E_{ab\;initio}$ are, respectively, the energies with PES-2020 and Level 2 for the same calculated points. The RMSE was 2.08 kcal mol^−1^, which was reasonable given the complex topography of the reactive system, where the appearance of deep wells in the entrance and exit channels made their correct location difficult.

### 4.2. Kinetics Results

#### 4.2.1. Rate Constant Calculations

There is an extensive literature regarding experimental temperature dependence for the title reaction. In 1981, Baulch et al. [9] reviewed the previous experimental data and showed that the rate constant was independent on temperature in the range of 300–700 K, with the expression k(T) of 1.66.10^−11^ cm^3^ molecule^−1^ s^−1^. Later, Sims and Smith [10,11] reported rate constants in the temperature range of 294–761 K, fitted to the expression k(T) of 1.52 10^−11^exp(1.50/RT), and at lower temperatures of 25–295 K, fitted to the expression k(T) of 2.77.10^−11^(T/298)^−1.14^, in cm^3^ molecule^−1^ s^−1^; to the best of our knowledge, this presents the only experimental evidence at low temperatures. They found that at temperatures T > 300 K, rate constants present little dependence with temperature, while at temperatures T < 300 K, rate constants strongly increase when temperature decreases. This behavior is characteristic of fast, barrierless reactions with a stabilized intermediate complex in the entrance channel. Finally, in 1995, Yang et al. [1] reported rate constants in the range of 294–716 K, fitted to the expression k(T) of 10^−11.02^ exp(254/T), in cm^3^ molecule^−1^ s^−1^, which are lower than those reported by Sims and Smith [10]. For instance, at 700 K, the values are 1.37.10^−11^ versus 1.97.10^−11^ cm^3^ molecule^−1^ s^−1^; i.e., differences of about 40%. This difference diminishes when the temperature decreases.

**Figure 4:**
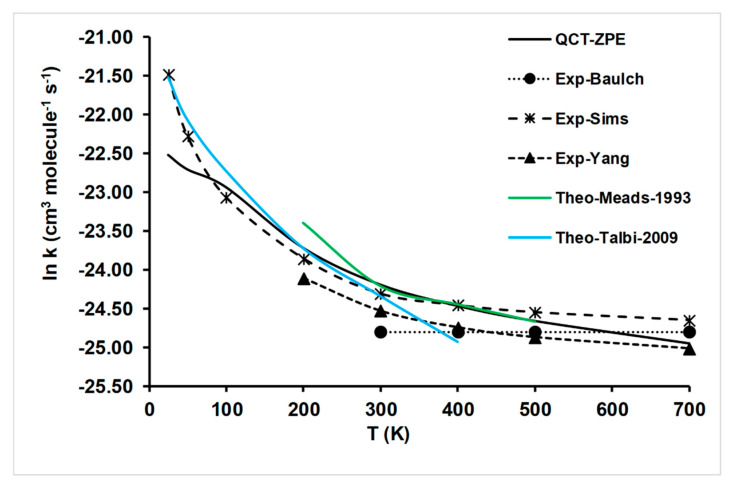
Rate constants (cm^3^ molecule^−1^ s^−1^) for the title reaction. Present results (quasi-classical trajectory (QCT)-ZPE) on the basis of our PES-2020. Solid black line; experimental review from [9], black circles; experimental measurements from [10,11], black crosses; experimental measures from [1], black triangles; theoretical results from [6]; solid green line; theoretical results from [7], solid blue line.

The QCT/PES-2020 rate constants obtained in the present work are listed in Table 3 and Figure 4, together with the experimental data and other theoretical results [6,7] for comparison. With respect to the experimental measurements, the widest temperature range was studied by Sims and Smith [10,11], at 25–761 K. The presented QCT rate constants on the PES-2020 surface simulated reasonably well the experimental evidence at temperatures T > 100 K, with agreement being worse at T < 100 K, where the QCT rate constants underestimated the experiments. In this low-temperature regime, the tunneling effect can be pronounced, and this quantum mechanics effect is not captured by QCT calculations. Nevertheless, in spite of the known limitations, rate constants calculations based on QCT/PES-2020 tools simulate reasonably well the experimental temperature dependence in the wide range of 25–700 K. In order to analyze this behavior at very low temperatures, quantum mechanical (QM) calculations are needed, which is beyond of the scope of the present work. However, we can analyze this problem based on our knowledge of atom–diatom reactions, where QCT and QM calculations have been compared. For instance, Lique et al. [44] and Teixidor and Varandas [45] analyzed the O + OH gas-phase reaction in the range of 10–350 K, which also presented a deep well in the entrance channel, by using QCT and QM (time-independent, TID) calculations based on the very accurate potential energy surfaces XXZLG [46,47] and CHIPR [45], respectively. Both PESs present different constructions, but they were fitted to >18,000 ab initio points. These studies showed, firstly, that even benchmark full QM calculations based on very accurate PESs underestimate the experimental rate constants, and secondly, that QCT rate constants are lower by a factor of about 2 than those predicted by QM calculations at low temperatures. Obviously, this QM/QCT difference at low temperatures is associated with quantum effects, zero-point energy conservation and tunneling effects. Therefore, based on these findings, we assume a similar behavior for the title reaction, and so the underestimation at very low temperatures obtained in the present work, QCT/PES-2020, will be corrected when QM calculations are performed.

In the comparison with other theoretical studies for the title reaction (also included in Figure 4), two other theoretical rate constants have been also reported [6,7]. Meads et al. [6] obtained rate constants in the temperature range of 200–500 K using the Rice-Ramsperger-Kassel-Marcus (RRKM) kinetic theory based on MP4SDQ//MP2 ab initio energies. The theoretical RRKM rate constants simulate the experimental data at T ≥ 300 K, but overestimate them at T = 200 K. Lower temperatures were not studied, but if one extrapolates them at T < 200K, the overestimation is noticeable. More recently, Talbi and Smith [7] calculated the minimum energy path at the CCSD(T)/6-311++G(3df,2pd) single-point level on optimized geometries at the CCSD/6-311G(d,p) level, obtaining the rate constants by Georgievskii and Klippenstein’s method [16], in the temperature range of 25–400 K. While in the range 25–300 K agreement with the experiment is excellent, at T > 300 K, the rate constants underestimate the experimental evidence. In summary, while the theoretical rate constants reported by Meads et al. failed at low temperatures, the results found by Talbi and Smith failed at high temperatures. In addition, note that in those previous studies, only a local description of the reactive system was calculated, in contrast with the global description presented in the present work. The discrepancies of these theoretical results, using different theoretical tools, with experiments show the difficulty in obtaining an accurate kinetics description of the title reaction, especially when a wide temperature range (25–700 K) is considered, thus emphasizing the importance of the results reported in the present study using a global potential energy surface.

#### 4.2.2. Kinetics Isotope Effects (KIEs)

KIEs represent a stringent test of several features of the topology of the new surface, such as the barrier height and width or ZPE near the dynamics bottleneck, and indeed of the quality of PES-2020. They are defined in the usual way; i.e., as the ratio of the rate constants for the unsubstituted (NH_3_) to the deuterated (ND_3_) reactions. To the best of our knowledge, only two experimental studies have been reported [1,6], and only one [1] reported a temperature dependence in the range of 221–740 K. At the common temperature, 296 K, the experimental KIEs differ: 1.89 [1] versus 2.01 [6]. The only theoretical study was reported by Meads et al. [6] in the temperature range of 200–500 K, concluding that the experimental KIEs were not predicted. For instance, at 300 K, the KIE was 0.95 versus the experimental value from the same group of 2.01 (296 K), thus showing the limitations of the theoretical tools used in that study. The QCT/PES-2020 KIEs calculated in the present work are listed in Table 4, together with experimental data for comparison. All KIEs are greater than 1—i.e., “normal”—and they increase with temperature, thus reproducing the experimental evidence. Given that the tunneling effect is negligible in this temperature range (200–700 K), the most important factor is the ZPE difference between NH_3_ and ND_3_ at the saddle point. So, for the same classical barrier, −1.45 kcal·mol^−1^, the adiabatic barriers, ∆H^≠^ (0 K), are −3.63 and −2.71 kcal·mol^−1^, respectively. This good agreement with experiments lends confidence to the PES-2020 developed in this work.

### 4.3. Dynamics Results

#### 4.3.1. Effect of the CN (v = 1) Vibrational Excitation on Reactivity

The only experimental dynamics property reported is the effect of CN(v) vibrational excitation on rate constants. Sims and Smith in 1988 [10] reported pulsed laser photolysis-laser-induced fluorescence measurements on the kinetics of CN (v = 0) and CN (v = 1) in the temperature range of 294–761 K. They found that vibrational excitation by one quantum increased the rate constant by a factor between 55% and 31% in this range. The QCT/PES-2020 results of the CN(v) vibrational excitation are listed in Table 5, together with the experimental evidence. The theoretical results show a slight increase in reactivity with vibrational excitation, in accordance with experiments, although they are lower. This increase in reactivity is associated with larger impact parameters with vibrational excitation and consequently to the opening of the “cone of acceptance” with CN (v = 1). The small increase found is due to the great reactivity of the vibrational ground-state, and so the increases in the maximum impact parameter, $b_{max}$, as shown in Equation (5), have little effect. In fact, the $b_{max}$ values are 13.5/14.5, 13.0/14.0 and 12.0/13.0 at 300, 500 and 700 K, respectively.

In order to understand this behavior, two approaches were used: (i) the coupling terms, B_i,F_(s), were used to measure the coupling of the mode i with the reaction coordinate F [48]; and (ii) the sudden vector projection (SVP) model [49,50,51], which also included translational and rotational couplings. The values of the couplings are shown in Table 6. Firstly, we observe that both approaches give a similar picture of the problem. Both approaches show the coupling of the CN stretching mode to the reaction coordinate, and therefore this mode promotes reactivity, in agreement with the experimental and theoretical results. Secondly, all vibrational modes in ammonia (stretching and bending) are coupled to the reaction coordinate, and therefore one could expect that vibrational excitation by one quantum of each mode would increase the reactivity. This issue has not been experimentally investigated. Finally, we observe that translational energy is more efficient at promoting reactivity than vibrational energy (the SVP values in Table 6) which is in accordance with Polanyi’s rules for “early” barrier reactions [52]. These last two theoretical predictions await future experimental confirmation.

#### 4.3.2. Effect of CN (v,j) Rotational Excitation on Reactivity

**Figure 5:**
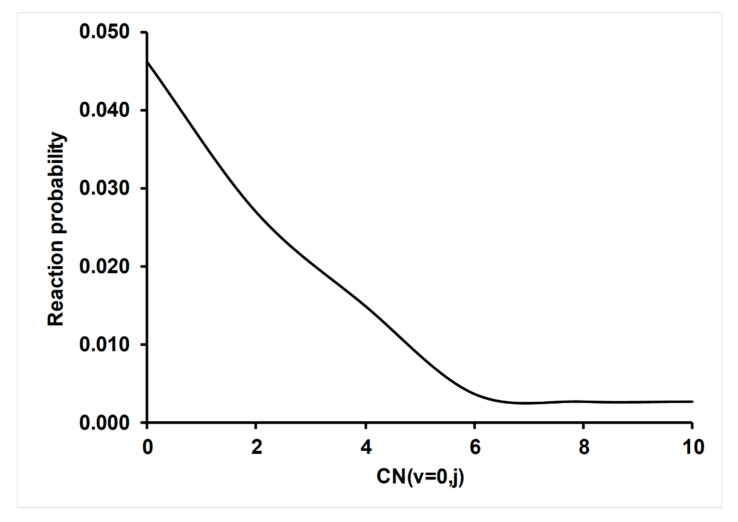
Reaction probability, P_r_ =N_r_/N_T_, as a function of the rotational quantum number, j, in the CN (v = 0,j) reactant. Theoretical results at T = 300 K using the QCT/PES-2020 tools.

In this section, we analyze the role of CN rotational excitation on reactivity, using as an example the temperature at 300 K (other temperatures show a similar tendency and are not presented here). Figure 5 plots the variation of the probability of reaction, P_r_ = N_r_/N_T_, Equation (5), as a function of the rotational quantum number, CN (v = 0, j = 0–10). This probability strongly decreases when j increases in the range of j of 0–6, while at the larger range of j of 8–10, the probability practically remains constant. We believe that when the CN reactant is rotationally excited, less time is necessary to obtain the adequate orientation for collision and therefore, fewer reactive trajectories are obtained. In summary, rotational excitation produces a disorienting effect [53,54,55,56] in the entrance channel, which in this particular reaction, with a deep well in this channel, was enhanced.

#### 4.3.3. Product Angular Distribution

**Figure 6:**
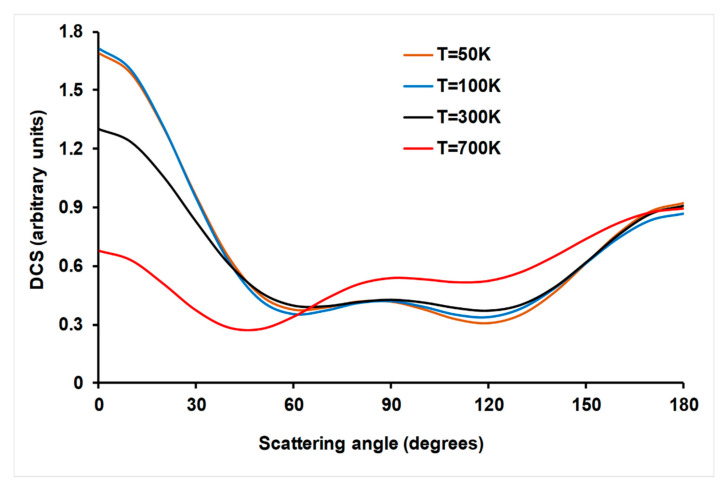
QCT/PES-2020 product angular distributions at different temperatures for HCN product (with respect to the incident CN).

Product angular distribution is an additional test of the existence of a complex mechanism for the title reaction. It is known that forward–backward angular scattering distributions are associated with the presence of persistent complexes along the reaction path [57]. The QCT/PES-2020 angular distributions of the HCN product with respect to the CN reactant were obtained as a differential cross section (DCS) which was fitted by the Legendre moment method [58]. The scattering angles at different temperatures are plotted in Figure 6. They show a forward-backward behavior at all temperatures, with a more forward tendency at low temperatures, which changes to a more backward tendency at higher temperatures. This behavior is associated to the presence of deep wells along the reaction path due to the presence of stabilized complexes. A similar forward–backward behavior was found by our group when studying the Cl(^2^P) + NH_3_ and OH + NH_3_ reactions [59,60], all which were characterized by persistent complexes in the entrance and exit channels. These wells produce a disorientation effect by which the initial trajectories lose the memory of the initial impact.

## 5. Conclusions

Based on a physical, intuitive description of the motions of nuclei in the reactive system of CN + NH_3_ → HCN + NH_2_—i.e., stretching and bending motions—an analytical global potential energy surface, named PES-2020, was developed for the first time for this six-body system. This surface depends on 33 adjustable parameters, it is symmetric with respect to the permutation of the three hydrogens in ammonia and it presents numerical gradients. High-level ab initio calculations were used to fit the surface describing all stationary points and the reaction path. Finally, this PES represents a noticeable improvement with respect to previous theoretical studies which only reported local surfaces of the reactive system using lower computational levels.

1. In general, PES-2020 simulates the properties (geometry, vibrational frequency and energy) of reactants, products and saddle points reasonably, showing an exothermicity of ∆H_r_(298K) = −20.05 kcal mol^−1^, reproducing the theoretical and experimental evidence, and a “submerged” barrier of −1.45 kcal mol^−1^, simulating the ab initio information used as input. However, due to the complicated topology of the title reaction, with deep wells in the entrance and exit channels, the description of the intermediate complexes is poorer. The stability, ∆H(0K), of the reactant complex is therefore underestimated by 1–2 kcal mol^−1^, while the stability of the product complex is overestimated by 3–4 kcal mol^−1^. Nevertheless, as in the case of the ab initio values used as a reference, PES-2020 also presents deep wells, and in the light of the results obtained, we believe that these differences are not determinant. Thus, PES-2020 represents a first approximation to the problem of the consequence of a compromise between time, efficacy and accuracy. Obviously, future theoretical studies will almost certainly improve the fitting, but we do not think that they will substantially modify the main conclusions obtained in the present work.
2. Based on PES-2020, a kinetics study was performed using QCT calculations. With respect to previous theoretical results, the rate constants obtained in the present work noticeably improve the agreement with the experimental evidence in the wide temperature range of 25–700 K. In addition, the experimental KIEs are reproduced for the first time. These comparisons represent a stringent test, and the good agreement with experiments lends confidence to the quality of PES-2020.
3. A dynamics study based on QCT/PES-2020 was also performed, for which experimental information for comparison was scarce. Firstly, vibrational excitation by one quantum of the CN (v = 1) reactant increased reactivity slightly with respect to the vibrational ground-state, by factors between 21 and 5% in the temperature range of 300–700 K, thus reproducing qualitatively the only experimental study. This behavior was explained by the coupling of this vibrational mode with the reaction coordinate. Secondly, CN(j) rotational excitation decreased reactivity. Finally, product angular distribution presented a forward–backward behavior, which is characteristic of reactions with deep wells in the entrance and exit channels. This tendency is additional proof of the existence of a complex mechanism for the title reaction. These last two effects, rotational excitation and angular distribution, can be explained by a disorientation effect in the entrance channel and so losing the appropriate orientation for effective collisions. These two theoretical predictions await experimental confirmation.

In summary, we present an exhaustive theoretical study of the title reaction, which includes the first analytical full-dimensional potential energy surface developed for this reactive system and a detailed kinetics and dynamics study. The comparison with available experimental evidence supports the quality of the theoretical tools used—QCT and PES-2020. Although the agreement is not yet quantitative, the present work represents a first step in this direction, and noticeably improves on previous theoretical studies.

## Figures and Tables

**Table 1:** Properties of reactants and products (distances in Å, frequencies in cm^−1^, energy in kcal mol^−1^) with PES-2020 and ab initio calculations ^a^.

Property	CN			NH_3_			HCN			NH_2_		
	PES	Level 1	Level 2	PES	Level 1	Level 2	PES	Level 1	Level 2	PES	Level 1	Level 2
**Geometry**												
C–N	1.172	1.175	1.180				1.172	1.157	1.165			
H-C							1.059	1.069	1.071			
<HCN							180.00	180.00	180.00			
N–H				1.014	1.014	1.017				1.028	1.026	1.031
<HNH				109.00	106.37	106.81				103.40	102.84	102.30
**Frequency**												
	2114	2103	2096	3684	3603	3621	3364	3434	3447	3553	3463	3458
				3684	3603	3621	2014	2118	2098	3252	3369	3363
				3312	3474	3486	756	715	713	1489	1547	1515
				1676	1676	1662	756	715	713			
				1676	1676	1662						
				1076	1085	1096						
**Energy**												
ZPE	3.02	3.01	3.00	21.60	21.61	21.65	9.85	9.98	9.96	11.86	11.98	11.58
∆E_R_	−17.51	−17.35	−18.41									
∆H_R_ (0 K)	−20.42	−19.89	−21.18									
∆H_R_ (298 K)	−20.05	−20.01	−21.0.6									

^a^ Level 1: Geometry, frequencies and energy at the explicitly correlated CCSD(T)-F12a/aug-cc-pVDZ level. Level 2: Geometry, frequencies and energy at the CCSD(T)/6-311++G(d,p) level. ZPE: zero-point energy.

**Table 2:** Other stationary point properties (distances in Å, frequencies in cm^−1^, energy in kcal mol^−1^) with PES-2020 and ab initio calculations ^a^.

Property	SP			RC2			PC		
	PES	Level 1	Level 2	PES	Level 1	Level 2	PES	Level 1	Level 2
**Geometry**									
N–H	1.031	1.020	1.022	1.018	1.012	1.012	1.032	1.025	1.029
N–H’	1.088	1.119	1.099	1.018	1.012	1.012	2.195	2.165	2.193
C–H’	1.512	1.485	1.539				1.062	1.078	1.080
C–N				3.197	2.000	2.060			
C–N(CN)	1.173	1.168	1.173	1.172	1.183	1.181	1.172	1.158	1.165
<NH’C	177.13	137.93	135.30				180.00	180.00	180.00
**Frequency**									
	3260	3551	3579	3604	3665	3652	3461	3485	3483
	3247	3450	3474	3604	3655	3648	3341	3391	3387
	2166	2184	2259	3239	3481	3486	3186	3297	3326
	1836	1922	2155	2115	2386	2107	2015	2108	2091
	1465	1594	1606	1659	1636	1621	1444	1551	1540
	1274	1461	1496	1659	1617	1617	769	858	889
	895	858	868	1071	902	996	769	831	875
	538	551	535	118	792	513	124	299	320
	516	387	360	118	603	455	87	226	250
	246	172	121	7118	456	360	84	149	148
	246	138	120	72	318	112	63	116	122
	810 i	878 i	697 i	72	243	91	58	87	107
**Energy**									
ZPE	20.99	23.30	23.70	24.95	27.75	26.55	22.02	23.44	23.65
∆E	−1.45	−1.62	−1.10	−8.12	−12.03	−11.16	−25.70	−23.00	−24.57
∆H (0 K)	−3.63	−2.94	−2.05	−7.80	−8.90	−9.27	−28.29	−24.17	−25.57

^a^ Level 1: Geometry, frequencies and energy at the explicitly correlated CCSD(T)-F12a/aug-cc-pVDZ level. Level 2: Geometry, frequencies and energy at the CCSD(T)/6-311++G(d,p) level.

**Table 3:** Rate constants (cm^3^ molecule^−1^ s^−1^) for the CN + NH_3_ reaction.

T(K)	QCT	Exp-1988 ^a^	Exp-1994 ^b^	Exp-1995 ^c^
25	1.65 × 10^−10^		4.7 × 10^−10^	
50	1.37 × 10^−10^		2.1 × 10^−10^	
100	1.09 × 10^−10^		9.6 × 10^−11^	
200	5.00 × 10^−11^		4.4 × 10^−11^	3.40 × 10^−11^
300	3.12 × 10^−11^	2.77 × 10^−11^		2.23 × 10^−11^
400	2.38 × 10^−11^	2.39 × 10^−11^		1.80 × 10^−11^
500	1.96 × 10^−11^	2.17 × 10^−11^		1.59 × 10^−11^
700	1.47 × 10^−11^	1.97 × 10^−11^		1.37 × 10^−11^

^a^ Experimental from [10]; ^b^ Experimental from [11]; ^c^ Experimental from [1].

**Table 4:** H/D kinetic isotope effects for the CN + NH_3_ reaction using the QCT/PES-2020.

T(K)	QCT	Exp.^a^	Exp.^b^
200	1.99	1.72	
300	2.02	1.89	2.01
500	2.10	2.04	
700	2.18	2.11	

^a^ Experimental values from [1]. ^b^ Experimental value at 296 K [6].

**Table 5:** QCT/PES-2020 and experimental ratios for CN (v = 1)/CN (v = 0). Reactivity at different temperatures.

T(K)	QCT	Exp. ^a^
300	1.21	1.55
500	1.16	1.40
700	1.05	1.31

^a^ Experimental values from [10].

**Table 6:** B_i,F_(s) and sudden vector projection (SVP) model coupling terms on the PES-2020 surface for the CN + NH_3_ reaction.

Species	Mode	Frequency ^a^	B_i,F_(s) ^b^	SVP
CN	Stretching	2114	0.66	0.085
NH_3_	Stretching	3684(d)	0.11	0.244
	Stretching	3313	1.74	0.230
	Deformation	1676(d)	2.05	0.260
	Deformation	1076	1.31	0.347
CN···NH_3_	Translation		-	0.795
	Rotation		-	0.118

^a^ Frequency in cm^−1^, where (d) means doubly degenerated. ^b^ The B_i,F_(s) values correspond to coupling in the entrance channel, s = −0.100 bohr.

## Data Availability

No applicable.

## Sample Availability

Samples of the compounds no available from the authors.
